# Precision immuno-oncology in NSCLC: integrating ADCs, therapeutic vaccines, and adoptive cell therapies for next-generation systemic treatment

**DOI:** 10.3389/fonc.2026.1904492

**Published:** 2026-08-14

**Authors:** Pankaj Garg, Sharad S. Singhal

**Affiliations:** 1 Department of Chemistry, GLA University, Mathura, Uttar Pradesh, India; 2 Department of Medical Oncology and Therapeutic Research, Beckman Research Institute of City of Hope, Duarte, CA, United States

**Keywords:** adoptive cell therapy, antibody-drug conjugates (ADCs), engineered immune cells, non-small cell lung cancer (NSCLC), precision immuno-oncology, therapeutic cancer vaccines

## Abstract

Targeted therapy and immune checkpoint inhibition have improved the treatment of non-small cell lung cancer (NSCLC); however, it remains one of the leading causes of cancer-related mortality worldwide. While all these therapeutic strategies have yielded clinical benefit in specific patient groups, resistance and tumor heterogeneity, short-term responses, and immune evasion remain current challenges in long-term therapeutic success. These constraints have led to the exploration of new precision immuno-oncology approaches for more specific targeting more efficient stimulation of antitumor immune responses and overcoming resistance mechanisms. Among these new modalities, antibody-drug conjugates (ADCs), therapeutic cancer vaccines, and adoptive cellular therapies are significantly changing the systemic treatment paradigm in NSCLC. Simultaneously, advances in neoantigen discovery, mRNA vaccine technology, and personalized vaccine design have renewed interest in therapeutic cancer vaccines for generating durable antitumor immune responses. In parallel, adoptive cellular immunotherapies, such as chimeric antigen receptor (CAR)-T cells, natural killer (NK)-cells, and tumor-infiltrating lymphocytes (TILs) are growing as effective approaches to improve immune-mediated tumor eradication in NSCLC. This manuscript summarizes the recent developments of these next-generation precision immuno-oncology platforms, highlighting their working mechanisms, progress in translation, and clinical use. It also explores strategies for combinational therapy, predictive biomarkers, inhibitors of tumor microenvironment modulation, and mechanisms of resistance in the context of therapeutic efficacy and patient selection. Collectively, these developing immunotherapeutic platforms as a whole constitute a brighter paradigm for more personalized and effective systemic treatment approaches in NSCLC.

## 1 Introduction

Non-small cell lung cancer (NSCLC) is the most common form of lung cancer and one of the most lethal cancer types globally. Although significant advances have been made in molecular diagnostics, targeted therapy, and immune checkpoint inhibitor (ICI) treatment of NSCLC in the last decade, the long-term clinical management of this disease remains a major challenge due to the heterogeneity of the disease, as well as the prevalence of therapeutic resistance, disease recurrence, and immune escape (1). Targeted agents that inhibit epidermal growth factor receptor (EGFR), anaplastic lymphoma kinase (ALK), and other oncogenic drivers have generated significant improvement in outcomes for subsets of patients, but for some patients, the response is either not achieved initially or is ultimately lost over time. In the same way, immunotherapy with agents targeting programmed death-1 (PD-1) and programmed death-ligand-1 (PD-L1) has changed the treatment of NSCLC, but this approach has proven effective only in a small proportion of patients and there is an urgent need for more effective and personalized systemic treatments (2).

Recent advancements in precision immuno-oncology have paved the way for the development of next-generation therapeutic platforms that not only increase tumor specificity but also further enhance antitumor immune responses and overcome resistance-associated mechanisms. Of these new therapeutic approaches, antibody-drug conjugates (ADC), therapeutic vaccines and adoptive cellular therapies are very promising for systemic therapy of NSCLC. Compared to traditional chemotherapies, ADCs are more selective in targeting tumor cells with highly potent cytotoxic drugs that deliver therapeutic agents to the targeted cell with minimal off-target effects. The ability of neoantigen identification, mRNA technologies and personalized vaccine design has also ushered therapeutic cancer vaccines into a new life with the goal of inducing long-term and tumor-specific immune activation (3). Other adoptive cellular immunotherapies, such as chimeric antigen receptor (CAR)-T cells, chimeric antigen receptor-based natural killer (CAR-NK) cells, and tumor infiltrating lymphocytes (TILs) are emerging as new immune therapies that could be beneficial for immune mediated tumor rejection in solid tumors, including NSCLC.

The adoption of these novel therapeutics, combined with precision medicine, biomarker-driven and combinational immunotherapeutic approaches, is slowly changing the face of the current treatment approach to NSCLC (4). Furthermore, the tumor microenvironment (TME) and its interaction with the immune system, and mechanisms of resistance are rapidly emerging areas of interest to exploit for better therapeutic response and patient classification (5). While these new therapeutic platforms show promising clinical promises on a single basis, their translation to the clinic has been highly variable and depends critically on the tumor type, immune resistance mechanisms, availability of the correct biomarker, tumor heterogeneity, and clinical maturity. The biological and clinical mechanisms underlying these differences are gaining significance in the development of future treatments, and in the selection of patients for NSCLC treatment. This article will provide a critical look at the biological and clinical drivers that have contributed to the differing success of the different precision immuno-oncology platforms in NSCLC, in contrast to previous descriptive reviews. Special attention is paid to the mechanisms underlying the efficacy of some therapeutic strategies while others have failed and the influence of tumor heterogeneity, resistance to therapy, choice of biomarkers, and translational barriers. This manuscript also highlights lessons learnt from failed clinical trials and identifies the next areas of research that will inform the next generation of precision immunotherapies. This perspective not only critically examines these novel therapeutic platforms but also considers them in the context of the changing clinical landscape of NSCLC, their current therapeutic applications, patient stratification on the basis of biomarkers, their clinical maturity in comparison to other platforms, and the realistic potential for incorporating these novel platforms into routine use in the clinic (3–5).

## 2 Evolution of precision immuno-oncology in NSCLC

### 2.1 Transition from conventional therapy to precision oncology

There was a great deal of transformation in the treatment of NSCLC in the past two decades. In the past, the most common systemic treatment available for advanced NSCLC was platinum drugs, which were often found to be ineffective due to lack of selectivity, systemic toxicity, and poor long-term survival benefits. Oncogenic driver mutations such as mutations in EGFR, ALK, ROS1, BRAF, MET, RET, and KRAS were first discovered in NSCLC, which initiated precision oncology (6). Targeted molecular drugs against these mutations have resulted in remarkable gains in response rates and progression free survival (PFS) in biomarker-selected patient populations. Despite this progress, the presence of acquired resistance, intratumoral heterogeneity, and disease progression remain significant therapeutic barriers and demand new systemic treatment strategies (7).

### 2.2 Emergence of immunotherapy in NSCLC

The advent of ICIs that target PD-1, PD-L1, and the cytotoxic T-lymphocyte-associated protein-4 (CTLA-4), has opened a new era in the treatment of NSCLC that promises the restoration of antitumor immunity. Advanced and metastatic NSCLC tumors showed positive results with pembrolizumab, nivolumab, atezolizumab, and durvalumab in high PD-L1 positive tumors (8). Immunotherapy also set the groundwork for use in combination treatment strategy, which combines chemotherapy, targeted therapy and radiotherapy. While these successes have been realized, the response only endures in a minority of patients; and the majority develop resistance via immune escape mechanisms, a lack of suitable tumor immunogenicity, T-cell exhaustion, and an immunosuppressive TME. This restriction has given rise to new generation of immunotherapeutic platforms which are more specific, resistant to resistance and provide improved long-term clinical benefit (9).

### 2.3 Need for next-generation systemic therapeutics

**Figure 1:**
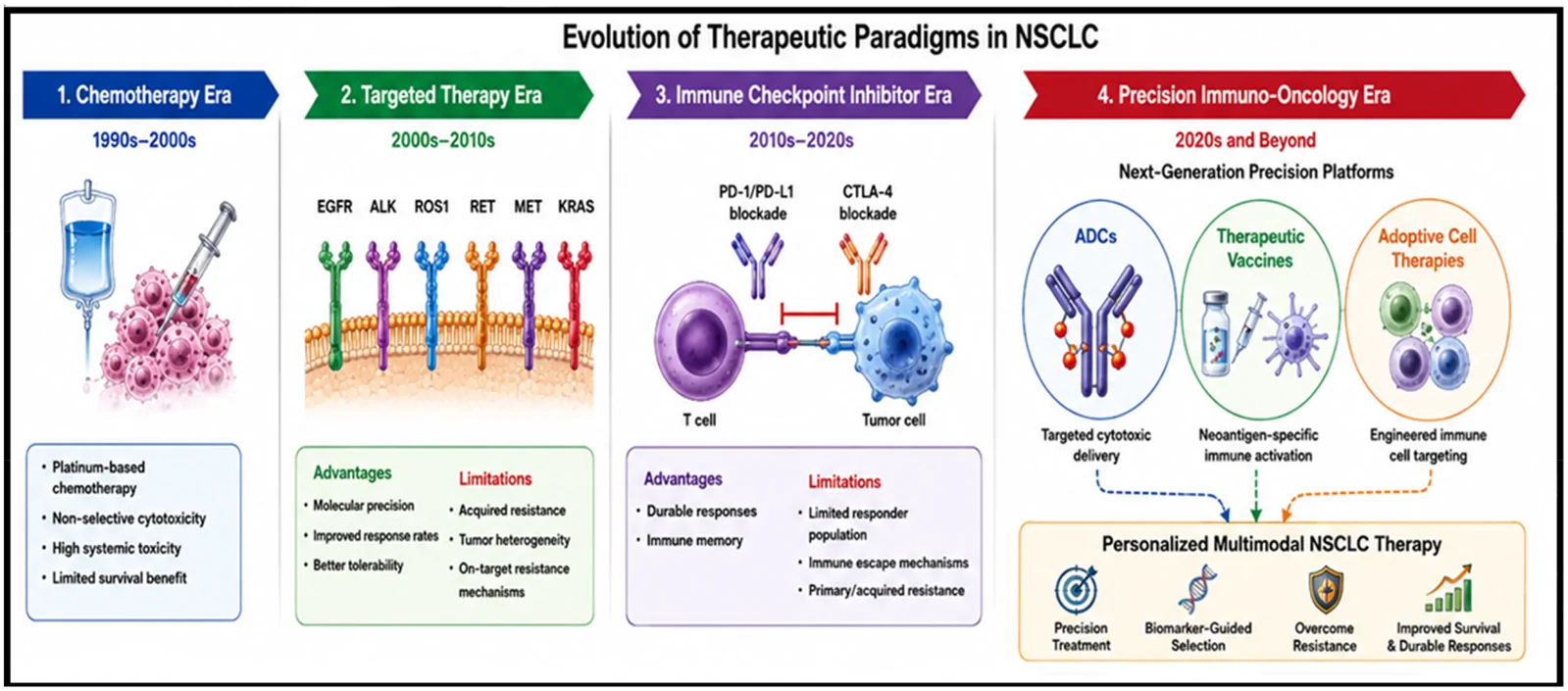
Conventional chemotherapy to precision immunotherapy: the evolution of therapeutic paradigms in NSCLC. This figure represents the stepwise shift in the treatment paradigms for systemic treatment of NSCLC over the last 30 years. During the chemotherapy period, platinum-containing regimens with known non-selective cytotoxic effects, toxic side effects, and only insignificant prolongation in survival were used. The next targeted therapy revolution led to a new class of treatments targeting oncogenic drivers including *EGFR, ALK, ROS1, RET, MET, and KRAS*, which led to better clinical results in molecularly defined cohorts of patients. Over the past few years, a major evolution in cancer immunology has been the revival of antitumor immunity by blocking PD-1/PD-L1 and CTLA-4, leading to significant increases in survival in certain patients. Inspired by these advances, the present precision immuno-oncology era combines next-generation therapeutic platforms such as ADCs, therapeutic cancer vaccines, and adoptive cellular therapies. These modalities allow for multimodal therapeutic strategies tailored to individual patients by biomarker selection, immune activation, new tumor targeting strategies, and potential for sustained clinical effects in NSCLC.

Traditional approaches such as targeted therapies and immunotherapies are inadequate to address the intricacies of NSCLC, necessitating novel therapeutic strategies. Multiple factors contribute to therapeutic failure, such as the tumor’s heterogeneity, dynamic genomic evolution, immune suppression, and adaptive resistance mechanisms. In this context, next-generation precision immuno-oncology strategies have become interesting alternatives capable of combining cytotoxicity against tumor cells with immune-mediated antitumor activity (10). ADCs are a class of compounds that provide the possibility of highly targeted delivery of cytotoxic therapeutic agents to tumor-associated antigens, thereby improving the precision of therapeutic action whilst reducing systemic toxicity. Likewise, therapeutic cancer vaccines attempt to induce tumor-specific and memory T cell responses by the activation of the immune system through neoantigen presentation. Engineered T cells and natural killer (NK) cells are also promising for their ability to eradicate tumors directly through immune- and cell-based mechanisms. These sophisticated systemic therapy approaches mark an important transition toward more personalized, biologically driven approaches to treating NSCLC (11). **Figure 1** summarizes the major therapeutic eras in NSCLC and highlights the emergence of ADCs, therapeutic vaccines, and adoptive cellular therapies as key components of next-generation precision immuno-oncology.

## 3 ADCs in NSCLC

### 3.1 Structural components and design principles of ADCs

ADCs are highly engineered pharmaceutical bioconjugates that are designed to incorporate the tumor-targeting specificity of monoclonal antibodies with the high cytotoxic activity of chemotherapeutic payloads. The three key components of an ADC include a monoclonal antibody (mAb) that binds to a tumor-associated antigen (TAA), a chemical linker, and a highly potent cytotoxic molecule. The antibody fragment allows for specific targeting of cancer cells with surface markers, whereas the linker portion affects the stability and activity of the released payload (12). Depending on the desired intracellular release mechanism, linkers can be cleavable or non-cleavable. Typical payloads are microtubule inhibitors or DNA-damaging drugs that are much more potent than standard chemotherapeutic drugs. Over the last few years, advances in linker chemistry, payload optimization, and antibody engineering have greatly enhanced the stability, therapeutic activity, and pharmacokinetics of ADCs in the treatment of NSCLC (13).

### 3.2 Mechanisms of antitumor action

The therapeutic activity of ADCs relies on a multi-step process that includes antigen recognition, binding, receptor-mediated internalization, intracellular trafficking, and payload release. Once the ADC binds to TAAs present on the surface of NSCLC cells, the ADC-antigen complex is internalized into the cells via endocytosis. Within the lysosomes, the linker is degraded or cleaved, resulting in the release of the toxic payload into the intracellular environment. These payloads then cause DNA damage, microtubule disruption, or cell death (apoptosis) in cancerous cells (14). Some ADCs also exhibit a bystander killing effect, in which free payload molecules spread into surrounding tumor cells lacking antigen expression, thereby increasing antitumor activity in heterogeneous tumors. Apart from direct cytotoxicity, some ADCs can also indirectly trigger immune activation through the induction of immunogenic cell death and modulation of the TME (15).

### 3.3 Clinically relevant ADC targets in NSCLC

In the context of NSCLC, numerous TAAs have proven to be valuable therapeutic targets for ADC development. Several targets have been widely studied, including human epidermal growth factor receptor 2 (HER2), trophoblast cell surface antigen 2 (TROP2), human epidermal growth factor receptor 3 (HER3), mesenchymal-epithelial transition factor (MET), and carcinoembryonic antigen-related cell adhesion molecule 5 (CEACAM5). HER2-directed ADCs have demonstrated promising activity in patients with HER2-mutant NSCLC, and TROP2-targeted ADCs are being actively investigated in patients with NSCLC, as TROP2 is expressed in many different subtypes of NSCLC (16). HER3-directed ADCs have shown therapeutic activity in clinical trials in EGFR-mutant tumors that are resistant to TKIs. Identification of the best antigen targets remains paramount for achieving therapeutic selectivity and avoiding off-target toxicity (17).

### 3.4 Approved and emerging ADCs in NSCLC

Recent clinical success has accelerated the incorporation of ADCs into the treatment landscape of NSCLC. The clinical development of these agents has been supported by several landmark early- and late-phase clinical trials that established clinically meaningful responses in biomarker-selected patient populations and progressively expanded the role of ADCs within thoracic oncology. Representative landmark clinical trials across the major precision immuno-oncology platforms discussed in this manuscript are summarized in **Table 1**.

**Table 1:** Representative landmark clinical trials and their clinical significance across emerging precision immuno-oncology platforms for NSCLC.

Therapeutic platform	Landmark trial	Clinical setting/target	Major findings	Clinical significance	References
ADC	DESTINY-Lung01	HER2-mutant NSCLC after prior therapy	Demonstrated durable objective responses and established proof-of-concept for HER2-directed ADC therapy	Supported regulatory approval of trastuzumab deruxtecan and established ADCs as a new therapeutic class in NSCLC	(16, 18, 59, 75)
	DESTINY-Lung02	HER2-mutant NSCLC	Confirmed efficacy and manageable safety at optimized dosing	Strengthened the role of trastuzumab deruxtecan in biomarker-selected patients	(16, 18, 59, 75)
	HERTHENA-Lung01	EGFR-mutant NSCLC after EGFR-TKI resistance	Demonstrated clinically meaningful activity of patritumab deruxtecan in resistant disease	Expanded ADC development beyond HER2 and supported HER3-targeted therapy	(17, 19, 59, 76)
Therapeutic Vaccine	MAGE-A3 Phase III	Resected MAGE-A3-positive NSCLC	Failed to improve disease-free survival	Demonstrated limitations of tumor-associated antigen vaccines and highlighted the importance of biomarker-guided patient selection	(25, 31, 67, 77)
	START (Tecemotide/L-BLP25)	Stage III NSCLC after chemoradiotherapy	No significant overall survival benefit in the overall population	Illustrated challenges related to immune tolerance and patient selection, informing next-generation vaccine development	(25, 29, 31, 67, 77)
CAR-T Cell Therapy	Early Phase I Clinical Studies	EGFR, HER2, Mesothelin, MUC1	Demonstrated acceptable feasibility and preliminary antitumor activity, but limited efficacy in solid tumors	Highlighted major barriers including poor trafficking, antigen heterogeneity, and T-cell exhaustion	(35, 38, 39, 44, 79)
CAR-NK Cell Therapy	Early Phase I Studies	Multiple solid tumor antigens	Favorable safety profile with encouraging early clinical activity	Supports development of off-the-shelf cellular immunotherapy but requires improved persistence	(4, 40, 41, 80)
TIL Therapy	Early Clinical Studies	Advanced solid tumors including NSCLC	Demonstrated feasibility of tumor-reactive lymphocyte expansion	Clinical benefit remains limited by manufacturing complexity and variable persistence	(42, 43, 46)
TCR-T Cell Therapy	Early Clinical Studies	Neoantigen/HLA-restricted intracellular targets	Demonstrated feasibility of targeting intracellular tumor antigens	Supports personalized precision immunotherapy but remains limited by HLA restriction and antigen escape	(44–46)

The pivotal DESTINY-Lung01 and DESTINY-Lung02 trials established trastuzumab deruxtecan as the first HER2-directed ADC to demonstrate clinically meaningful and durable responses in previously treated HER2-mutant NSCLC, ultimately leading to regulatory approval. Likewise, the HERTHENA-Lung01 study demonstrated encouraging activity of patritumab deruxtecan in patients with EGFR-mutant NSCLC following resistance to EGFR tyrosine kinase inhibitors, further supporting the expanding role of biomarker-selected ADC therapy. The antitumor activity of datopotamab, deruxtecan, and Sacituzumab govitecan against TROP2 has been encouraging in heavily pretreated patients with NSCLC (18). Likewise, telisotuzumab vedotin, which targets MET overexpression, has demonstrated therapeutic activity in selected patient populations. Current clinical trials continue to evaluate new ADC platforms, combining ADCs with other therapies such as ICIs and targeted agents, as well as exploring dual-payload approaches. Although several ADCs have demonstrated encouraging clinical activity, their therapeutic benefit has not been uniformed across different molecular subgroups. Early clinical experience indicates that treatment efficacy is strongly influenced by antigen biology, biomarker-guided patient selection, and toxicity profiles, emphasizing that biological context is as important as the ADC construct itself (19).

### 3.5 Resistance mechanisms associated with ADCs

Despite promising clinical results, resistance to ADC therapy remains a challenge in the management of NSCLC. Multiple biological mechanisms contribute to therapeutic failure, including alterations in antigen expression, intracellular trafficking, lysosomal processing, payload sensitivity, and tumor heterogeneity (20). Additionally, altered DNA repair mechanisms and defective apoptotic pathways may further reduce susceptibility to ADC-mediated cytotoxicity. Understanding these resistance mechanisms is essential for optimizing ADC design, improving biomarker-guided patient selection, and developing effective combination therapeutic strategies (21).

The increasing evidence for resistance to ADC therapy is not necessarily single-agent and more likely reflects dynamic evolution of the tumor during ADC therapy. Reduced expression of target antigen may lead to decreased ADC binding, and inefficient receptor internalization and altered trafficking pathways can limit transport of cytotoxic payloads to the lysosome. Further, acquired resistance to payload-mediated mechanisms of cytotoxicity and lysosomal dysfunction as well as upregulation of multidrug-efflux transporters can further limit therapeutic effectiveness. Another challenge is intratumoral antigen heterogeneity due to the fact that antigen-negative tumor cell populations can gain a survival advantage despite bystander killing. The observations suggest that overcoming ADC resistance will require integrated approaches, in which every component, such as the selection of patients based on biomarkers, the use of next-generation ADC engineering and rational combination therapies that are able to address multiple resistance pathways at the same time. Together, these findings make the point that biological resistance, not just cytotoxicity, is the key to overcoming resistance in a subset of patients and will be critical to future development of ADC therapy in NSCLC (20–22).

### 3.6 Challenges and toxicity considerations

Toxicity remains a clinically important consideration for ADC therapy, despite the greater selectivity of these agents compared with conventional chemotherapy. Several ADCs have shown adverse effects in NSCLC patients, such as interstitial lung disease (ILD), pneumonitis, hematological toxicities, hepatotoxicity, and gastrointestinal adverse effects. Off-target toxicity may result from premature release of the payload, uptake in non-antigen-expressing tissues, or low-level expression of the antigen in normal tissues. Moreover, antigen heterogeneity and variable biomarker expression hinder patient stratification and prediction of therapeutic response (22). Overcoming these needs will demand ongoing development and optimization of ADC engineering and enhanced clinical monitoring during therapeutic development. However, in clinical practice, further increase in the cytotoxic effect does not result in improvement of therapeutic effect. Early findings from clinical trials revealed some encouraging signs of potential efficacy, others proved to be more heterogeneous than anticipated and dose limiting toxicities were observed and adverse events were often seen in the lungs. In addition, toxicities of treatments are still strongly dependent on the linker chemistry, payload properties, and tissue expression of the targets. For instance, the ILD seen with some of the Topoisomerase I-based ADCs has become a clinically relevant side effect with the need to recognize and monitor these patients closely. In addition, there are no standardized assays for specific biomarkers and dynamic temporal changes in the expression of antigens during treatment, which makes patient selection and contributes to clinical heterogeneity. All these obstacles underscore the need for the validation of biomarkers, the optimization of drug toxicity management, and the precise selection of patients for successful clinical implementation of ADCs (16, 22).

### 3.7 Future directions in ADC engineering

Developing next-generation ADCs is a key area of focus in the search for greater therapeutic precision, lower toxicity, and the circumvention of resistance-associated limitations. Bispecific ADCs that target multiple tumor antigens, dual-payload ADCs for overcoming tumor heterogeneity-associated resistance, and conditionally activated ADCs that respond to the TME are emerging strategies. The optimization of ADCs is also being advanced by new technologies in the fields of artificial intelligence (AI)-driven drug design, computational modelling, and biomarker identification. Moreover, novel linker technologies and immune-stimulatory payloads are being investigated to enhance tumor immune activation and long-term efficacy in NSCLC (23).

Three key factors have been crucial for clinical success of ADCs in NSCLC: proper selection of antigens, enhanced linker chemistry, and optimization of potent cytotoxic payloads. Remarkable clinical results have been obtained with HER2-directed ADCs largely because HER2 is a biologically validated target, with biomarker-defined eligibility criteria and internalization characteristics (16, 18). However, the clinical results of ADCs targeting more heterogeneous antigens have been relatively heterogeneous, likely due to antigens that are not equally expressed across patient populations, acquired antigen resistance, and treatment-related toxicities, such as interstitial lung disease (18, 22). These clinical experiences also illustrate that higher the cytotoxic potency, higher the price that must be paid, so that it is not enough to increase the potency of chemotherapy. Rather, the key to further ADC development is selection of biomarker targets, ensuring a tight therapeutic window, new payload technologies, and rational combination approaches that will overcome the resistance and heterogeneity of tumors. Taken together, these developments show that integration of strong biological knowledge in the form of robust biological target validation with precise patient selection and not only advances in drug engineering will ultimately lead to successful clinical translation. While therapeutic cancer vaccines and adoptive cellular therapies are still largely investigational, the use of ADCs is gaining increasing acceptance into routine clinical use. Clinically, the newly developed precision immuno-oncology platforms mentioned in this manuscript are the most well developed among all the others, and this is the case for the ADCs. Their primary indication is changing in patients with advanced NSCLC with progression after standard platinum-based chemo-immunotherapy or in those where targeted therapy is indicated and beneficial, especially in patients with HER2 mutations or HER3 expressing tumors, who are good candidates for targeted therapy. Further phase II/III clinical trials will assess the use of ADCs in earlier lines of therapy and as first-line combination therapy. In the emerging immunotherapeutic platforms detailed in this manuscript, ADCs are now by far the closest to routine clinical use in NSCLC (22, 23).

## 4 Therapeutic cancer vaccines in NSCLC

### 4.1 Immunological basis of cancer vaccination

Therapeutic cancer vaccines activate and amplify the immune response against tumors, enabling the immune system to eliminate tumor cells. Therapeutic cancer vaccines seek to recruit and stimulate cells that can kill tumor cells, especially cytotoxic T lymphocytes (CTLs), but do not produce protective immunity against infectious diseases. TAAs and tumor-specific neoantigens resulting from somatic mutations are key targets for vaccine development in NSCLC. After recognizing antigens presented by major histocompatibility complex (MHC) molecules, activated T cells proliferate and travel to tumor tissue to mediate antitumor activity (24). Several factors contribute to therapeutic vaccination success, such as selecting the appropriate antigen, the immunogenicity of the vaccine, efficient antigen presentation, and overcoming the immunosuppressive TME. Recent progress in tumor genomics and immune phenotyping has greatly improved the ability to identify immunogenic neoantigens, enabling more precise and personalized cancer vaccine approaches for NSCLC (25).

### 4.2 Types of therapeutic vaccines

There are several different platforms of therapeutic vaccines studied in NSCLC involving several different methods of delivering antigens, the likelihood of inducing immunity and ease of manufacture. Peptide-based vaccines contain short fragments of antigen that trigger T-cell immune responses to that antigen. These vaccines have a relatively simple production process and high safety properties; however, the induction of high immunogenicity requires using the proper adjuvants or combination with immunogenic agents (26). Vaccines based on dendritic cells are loaded with tumor antigens to sensitize T cells in the reinfused patient. DNA and RNA vaccines have multiple advantages, such as the ability to carry several tumor antigens in one vaccine and their potential for rapid production. Viral vector vaccines are based on engineered viruses containing tumor antigens, which result in robust cellular immune responses. In recent years, precision medicine has expanded to the development of personalized neoantigen vaccines, which are generated via next-generation sequencing and computer algorithms that identify and predict neoantigens, thereby providing more effective targeting of patient-specific mutations involved in tumor development (27).

### 4.3 mRNA vaccine platforms in NSCLC

Messenger RNA (mRNA) vaccines are gaining momentum in the prevention of infectious diseases and are now the focus of research for NSCLC. The working mechanism of mRNA vaccines is that they deliver mRNA containing TAAs and/or neoantigens to host cells, which translate these antigens and elicit an adaptive immune response. Lipid nanoparticle-based delivery systems have significantly enhanced the stability of mRNA, the efficiency of intracellular delivery, and vaccine immunogenicity. One way for this to happen is with the use of personalized mRNA vaccines derived from genomic sequencing of patients’ own tumors to specifically target unique neoantigens generated through mutations. This personalized treatment has several benefits in overcoming tumor heterogeneity and minimizing off-target immune responses (28). Furthermore, mRNA vaccines can be rapidly designed and produced and offer considerable flexibility in targeting novel therapeutic molecules, making them attractive candidates for precision oncology. With these benefits, there are some problems as well, chief among them being limited vaccine persistence, the TME’s immunosuppressive properties, and the need for optimal antigen selection.

### 4.4 Clinical progress of NSCLC vaccines

Several therapeutic vaccine platforms are now entering clinical evaluation, and there has been a significant increase in clinical investigations of therapeutic vaccines in NSCLC over recent years. To elicit survival benefits through antibody-mediated EGF depletion and inhibition of EGFR signaling pathways, a vaccine based on epidermal growth factor (EGF) was evaluated in selected NSCLC patients and found to be encouraging. A viral vector vaccine targeting mucin-1 (TG4010) has demonstrated positive effects when used in conjunction with chemotherapy and ICIs (29). Early clinical trials with neoantigen vaccines have also shown the ability to elicit strong T-cell responses and to increase the number of tumor-reactive immune cells. In addition, current clinical studies are evaluating mRNA vaccines, dendritic cell vaccines, and multi-antigen vaccine platforms alone or in combination with PD-1/PD-L1 inhibitors. While a variety of vaccine approaches are in the early stages of development, emerging clinical evidence suggests that these vaccines can be used to boost antitumor immunity and therapeutic responses in patients with NSCLC (30). While some of the previously tested therapeutic vaccine regimens were immunogenic, none provided statistically significant improvements in overall survival or long-term clinical benefit at phase III. For instance, vaccines aimed at *MAGE-A3* and *tecemotide (L-BLP25)* were expected to show strong promise initially, however, did not ultimately yield clinically meaningful results, partly due to immune tolerance, inappropriate patient selection, tumor heterogeneity and lack of induction of long-term antitumor immunity. All of these experiences have significantly changed the landscape of the field by highlighting the need for biomarker-driven patient selection, rational combination therapy with ICIs instead of immune vaccine alone, and the identification of the neotargets to be targeted (25, 31).

### 4.5 Challenges limiting vaccine efficacy

Although cancer vaccine technologies have advanced, there are many hurdles to overcome with therapeutic cancer vaccines in NSCLC. The highly immunosuppressive TME, defined by regulatory T cells (Tregs), myeloid-derived suppressor cells (MDSCs), inhibitory cytokines, and tumor immune checkpoint signaling pathways, all contribute to poor T-cell activation and persistence and represent a major obstacle. Furthermore, tumor heterogeneity and antigen loss decrease the effectiveness of vaccines by allowing immune escape and promoting differences in the recognition of different tumor cell populations (31). Moreover, some TAAs may lack sufficient immunogenicity, leading to suboptimal activation of the immune system. The development of immune tolerance toward self-antigens, as well as the absence of infiltration of activated immune cells into tumor tissues, are also factors associated with therapeutic resistance. Furthermore, variability in patient immune status, antigen selection, and vaccine administration approaches exacerbates the challenges of optimizing vaccine-based therapies in NSCLC (32). Together these biological barriers account for much of the poor success of many early therapeutic cancer vaccine studies to demonstrate meaningful overall survival or durable clinical benefit from encouraging preclinical results and immunogenicity. These findings underscore the importance of designing the right vaccine, as well as patient stratification based on biomarkers and approaches to combat the immunosuppressive tumor microenvironment for optimal vaccine therapy.

### 4.6 Combination strategies to enhance vaccine responses

Cancer vaccines are increasingly being combined with other systemic treatment modalities that stimulate the immune system and overcome tumor-induced immune suppression to achieve greater therapeutic efficacy. Combination treatments with therapeutic vaccines and ICIs have been particularly promising, as they promote the initiation of antigen-specific T-cell activation and inhibit T-cell exhaustion. The combination of vaccines with chemotherapy or radiotherapy can further enhance the tumor response through immunogenic tumor cell death and antigen release (33). The combination of vaccines with ADCs and targeted therapies is also being investigated to further improve their immunogenicity and increase immune recognition. Furthermore, studies are underway to enhance the durability of the immune response and the precision of therapy using adjuvant systems, cytokine modulation, and personalized vaccine platforms. All these combinational strategies have the potential to further improve clinical outcomes and facilitate the implementation of more effective precision immuno-oncology strategies in NSCLC (34).

While therapeutic cancer vaccines are among the most appealing approaches in precision immune oncology, their clinical translation has proven much more difficult than initially hoped. Despite promising immunogenicity, several early vaccine platforms showed only slight survival benefit due to immune tolerance, poor choice of antigens, tumor heterogeneity, and the hyper immunosuppressive TME. Such experiences have led to a paradigm change from universal vaccine strategies to personalized neoantigen based and mRNA vaccine platforms combined with patient selection based on biomarkers and immune checkpoint blockade [25.31]. Thus, rational combination strategies, personalized vaccine antigen selection and efficient modulation of tumor immune microenvironment are likely to play a more important role in the success of therapeutic cancer vaccines in the future than the formulation itself. Therapeutic cancer vaccines are in the investigational stage and are not likely to be used as standalone therapies for NSCLC in the near future, from a clinical perspective. Rather, their potential to become effective drugs in the clinic seems to be in synergy with ICIs and other immunomodulatory agents, especially in patient subsets selected based on tumor markers that are associated with immunologic responsiveness. The widespread use of their incorporation to clinical routine will require the proof of significant improvements in survival and long-term clinical benefit in properly designed randomized clinical trials (31).

## 5 Adoptive cell therapies in NSCLC

### 5.1 Principles of adoptive cellular immunotherapy

Adoptive cellular immunotherapy is a cutting-edge approach in the field of immuno-oncology that involves collecting, engineering, expanding, and re-introducing immune cells into the patient to boost antitumor immunity. In contrast to conventional immunotherapies that require the stimulation of the patient**’**s own immunity, adoptive cell therapies deliver patients functionally improved immune effector cells, which can directly target and kill tumor cells. This is especially appealing in the context of NSCLC because the tumor is likely to have a high mutational burden, and tumor-associated neoantigens may be available to serve as targets for the immune system (35). Immune cell engineering performed *ex vivo* can enhance tumor recognition, immune cell cytotoxicity, persistence, and resistance to immunosuppressive signals by changing the genetic characteristics of T cells or NK cells. The overall treatment comprises the collection of immune cells and their activation, genetic modification, and culture expansion in the laboratory, followed by the subsequent reinfusion of the modified cells into the patient following lymphodepleting conditioning therapies (36). There are multiple challenges to successfully targeting adoptive cell therapies to tumor antigens. Conventional immune priming is not required for engineered immune cells to selectively recognize TAAs, which are often used to evade the immune system in NSCLC. In addition, major advances in cellular engineering, synthetic biology, and gene-editing technologies have significantly increased the capacity to modify and improve the function of immune cells without increasing toxicity and increase the specificity of therapeutic activity. While adoptive cellular immunotherapy has shown great success in hematological malignancies, further research is ongoing in solid tumors, including NSCLC, where the TME is more complex, and immune cell infiltration and persistence are more difficult (37).

### 5.2 CAR-T cell therapy in NSCLC

One of the most well-studied adoptive cellular immunotherapy strategies is chimeric antigen receptor (CAR) T-cell therapy. CAR-T cells are genetically modified T cells that possess synthetic receptors that recognize TAAs in an MHC-independent fashion. CARs are composed of three sections: an extracellular antigen-binding domain, a transmembrane domain, and intracellular signaling domains, which are essential for T-cell activation and proliferation. Upon binding to antigens, CAR-T cells are activated, release cytokines, proliferate, and kill tumor cells directly via the secretion of cytokines, perforin, and granzymes. Several TAAs have been investigated as CAR-T targets in NSCLC, such as EGFR, mesothelin, HER2, mucin-1 (MUC1), carcinoembryonic antigen (CEA), and PD-L1. CAR-T cells have exhibited promising preclinical antitumor activity in preclinical models of NSCLC, and preliminary clinical effectiveness and safety have been observed in early-phase clinical trials (38).

However, unlike the remarkable clinical success achieved in hematological malignancies, early clinical studies in NSCLC have generally produced modest response rates and limited durability of benefit. These findings indicate that simply translating CAR-T technology from hematologic cancers to solid tumors is insufficient without simultaneously overcoming antigen heterogeneity, poor tumor trafficking, immune suppression, and T-cell exhaustion. The effectiveness of CAR-T therapy in NSCLC has not been as dramatic as that in hematological cancers, however. This limitation is due to several hurdles, such as heterogeneous antigen expression, poor trafficking of CAR-T cells to tumor tissues, immunosuppressive signaling in the TME, and T-cell exhaustion. Also, severe toxicities, including cytokine release syndrome (CRS) and immune-related adverse events (irAEs), continue to be clinical issues that require therapeutic monitoring and management (39). These findings indicate that, unlike in hematological malignancies, the major limitation of CAR-T therapy in NSCLC is not the engineering of immune cells alone but the inability to overcome the complex biological barriers imposed by solid tumors.

### 5.3 CAR-NK cell therapy

CAR-NK cell therapy has become a promising alternative to CAR-T therapy in treating solid tumors such as NSCLC. NK cells are innate immune effectors that have the ability to recognize and destroy malignant cells in the absence of prior antigen sensitization. CAR-NK cells are genetically modified NK cells that possess chimeric antigen receptors, which improve target specificity while maintaining the inherent cytotoxic functions of NK cells (40). Unlike CAR-T cells, CAR-NK therapies have several potential benefits, such as a decreased risk of cytokine release syndrome, a reduced risk of graft-versus-host disease (GVHD), and improved safety profiles. One additional benefit of CAR-NK therapy is the possibility of off-the-shelf production using donor-derived or induced pluripotent stem cell (iPSC)-derived cells. This method could solve numerous logistical and manufacturing problems associated with autologous CAR-T therapies, such as the extended production time and quality fluctuations in patient-derived immune cells. CAR-NK cells can also kill tumor cells via both CAR-dependent and natural cytotoxic mechanisms, which may enhance tumor-killing efficacy in heterogeneous NSCLC tumors. The clinical effectiveness of CAR-NK therapies in NSCLC, however, needs to be proven in long-term. They are limited in their therapeutic activity by very short duration of *in-vivo* persistence, their inability to expand into the tumor mass, their inability to penetrate into the tumor tissue, and their inability to overcome the immunosuppressive TME. Moreover, the ability to induce long-lasting anti-tumor effects in solid tumors is not yet well established in the clinic, requiring further tuning before being broadly available. Thus, current engineering strategies aim at further optimizing cytokine stimulation, metabolic fitness, tumor infiltration, and persistence, in combination with other forms of immunotherapy, to achieve durable clinical benefit in NSCLC (41).

### 5.4 Tumor-infiltrating lymphocyte therapy

TIL therapy is a process of *ex vivo* expansion and reinfusion of naturally occurring lymphocytes that react to tumor antigens extracted directly from patient tumor tissue. TILs are immune cells that specifically recognize TAAs present within the TME and therefore are strong candidates for personalized cellular immunotherapy. Following tumor resection and/or biopsy, TIL populations are isolated and expanded in cytokine-containing culture media before their reinfusion into patients, sometimes with the addition of lymphodepleting chemotherapy and interleukin-2 support. TIL therapy has been shown to be clinically very effective in melanoma and is now being explored in NSCLC because of the relatively high number of neoantigens induced by smoking-related mutations (42). The use of single-antigen-targeting strategies is associated with immune escape, which is less likely to occur with the broad antigen specificity of expanded TIL populations, given their recognition of multiple TAAs and neoantigen targets. Furthermore, TIL therapy could be beneficial in heterogeneous tumors containing more than one antigenic population. However, there are still several hurdles to the widespread use of TIL therapy in NSCLC, such as low frequencies of functional lymphocytes, variable expansion efficiencies, limited persistence of TILs following infusion, and an immunosuppressive TME. Nevertheless, the therapeutic potential of TILs continues to be enhanced by improvements in TIL selection, expansion protocols, and combination therapies (43).

### 5.5 TCR-engineered T cell therapies

Another novel adoptive immunotherapy strategy is the modification of T cells with T-cell receptors (TCRs) that specifically recognize intracellular peptides derived from tumors and presented in the context of MHC molecules. In contrast to CAR-T cells, which specifically target cell-surface antigens, TCR-engineered T cells have the potential to target both neoantigens and intracellular oncogenic proteins, which represents a significant increase in the number of potential therapeutic targets in NSCLC (44). An important advantage of TCR-engineered therapies is the targeting of specific antigens, particularly those derived from tumor-specific mutations that generate highly immunogenic peptides not naturally expressed in normal tissues. The ability to identify personalized TCR targets has improved for NSCLC patients with advances in next-generation sequencing and computational neoantigen prediction. However, TCR-based therapies still rely on human leukocyte antigen (HLA)-restricted antigen presentation, which restricts their application to patients with compatible HLA alleles. In addition, therapeutic effectiveness may be diminished because of tumor antigen loss, impaired antigen presentation, and immune suppression within the TME. As a whole, TCR-based cellular immunotherapies have potential as precision immunotherapies to target intracellular oncogenic drivers and patient-specific neo-antigens in NSCLC (45). Despite their therapeutic promise, the translation of these therapies is still at its incipient stage and currently limited by restricted HLA compatibility, tumor antigen heterogeneity, immune escape, poor T-cell persistence, and the highly immunosuppressive TME, among other issues. Therefore, better identification of neoantigens, more general applicability of the HLA, greater T cell fitness, and rational combination strategy to circumvent TME resistance will be required for successful clinical implementation.

### 5.6 Barriers to cellular therapy in NSCLC

Adoptive cellular therapy is an important innovation in precision immuno-oncology, but there remain a few biological and clinical challenges that hinder the use of adoptive cellular therapies in patients with NSCLC. A highly immunosuppressive TME, characterized by Tregs, tumor-associated macrophages (TAMs), MDSCs, inhibitory cytokines, hypoxia, and metabolic competition, is one of the most important barriers. All these factors work together to reduce immune cell activation, proliferation, and tumor-killing capacity (46). The biggest hurdle, however, is the state of T-cell exhaustion. In chronic inflammatory conditions, continuous antigen stimulation can result in loss of function and increased expression of inhibitory receptors, including PD-1, TIM-3, and LAG-3. Furthermore, poor trafficking and infiltration of engineered immune cells into tumors limit the effectiveness of these therapies in solid malignancies. NSCLC tumors are further resistant to immune cell infiltration due to dense stromal architecture, abnormal vasculature, and chemokine signaling imbalances. In addition, adoptive cellular immunotherapies continue to present several safety concerns, such as cytokine-related toxicities, including cytokine release syndrome and neurotoxicity. Manufacturing complexity, treatment costs, and variability associated with patient immune status are additional translational hurdles that must be overcome to achieve widespread clinical applicability (47). Together, these biological and translational barriers account for the high degree of clinical success of adoptive cellular therapies in hematological malignancies that have not yet proven to be reproducible in NSCLC. Moving forward, more powerful tools for immune-cell engineering, as well as strategies to overcome the immunosuppressive TME, increase in immune-cell trafficking and persistence, and development of robust predictive biomarkers, will be vital in enabling adoptive cellular therapies to lead to long-lasting clinical responses, in addition to the development of complementary immunotherapeutic strategies that can have long-lasting clinical impacts.

### 5.7 Emerging engineering strategies

Next-generation adoptive cellular therapies have recently been developed through advances in immune engineering and synthetic biology and may overcome many of the therapeutic limitations associated with NSCLC. Armored CARs are genetically engineered CAR-T cells that express built-in cytokines or stimulatory molecules, allowing for enhanced immune persistence and resistance to tumor-induced immunosuppression. Multi-target CARs capable of recognizing multiple TAAs are being designed to minimize antigen escape and enhance therapeutic coverage in heterogeneous tumors (48). The opportunities provided by gene-editing technologies, particularly CRISPR/Cas-based systems, have also expanded significantly for enhancing immune cell function, as they can be used to modify inhibitory pathways, endogenous receptors, and immune checkpoint signaling mechanisms. Engineering programmable immune circuits, controllable activation systems, and logic-gated cellular therapies that are selectively activated within the TME are also being actively explored. Furthermore, metabolic reprogramming, cytokine engineering, and combination therapy with ICIs are currently being investigated to improve immune cell persistence and antitumor efficacy (49). These new engineering breakthroughs are likely to greatly enhance the precision, safety, and therapeutic benefit of adoptive cell therapies for NSCLC. A comparative overview of the major next-generation precision immuno-oncology platforms currently under investigation in NSCLC, including ADCs, therapeutic cancer vaccines, and adoptive cellular therapies, is summarized in **Table 2**.

**Table 2:** Comparison of next-generation precision immuno-oncology platforms in NSCLC.

Therapeutic platform	Mechanism of action	Major targets/components	Key advantages	Major limitations	Representative examples	References
Antibody–Drug Conjugates (ADCs)	Targeted delivery of cytotoxic payloads following antigen recognition and internalization	HER2, HER3, TROP2, MET, CEACAM5	High tumor specificity, potent antitumor activity, reduced systemic toxicity, bystander killing effect	Antigen heterogeneity, payload resistance, interstitial lung disease, off-target toxicity	Trastuzumab deruxtecan, Patritumab deruxtecan, Datopotamab deruxtecan, Telisotuzumab vedotin	(12, 16, 19, 59, 66, 76)
Therapeutic Cancer Vaccines	Activation of antigen-specific adaptive immune responses through tumor-associated antigens or neoantigens	Peptide antigens, neoantigens, mRNA, dendritic cells, viral vectors	Personalized immune activation, immune memory formation, favorable safety profile	Limited immunogenicity, immune suppression, antigen loss, variable patient responses	CIMAvax-EGF, TG4010, personalized neoantigen vaccines, mRNA vaccines	(25, 28, 30, 31, 67, 77, 78)
CAR-T Cell Therapy	Genetic engineering of T cells expressing chimeric antigen receptors for tumor recognition	EGFR, HER2, MUC1, Mesothelin, CEA	Highly specific tumor targeting, potent cytotoxicity, long-term immune surveillance	Poor trafficking, T-cell exhaustion, cytokine release syndrome, antigen escape	HER2-CAR-T, Mesothelin-CAR-T	(35, 38, 39, 44, 48, 79, 80)
CAR-NK Cell Therapy	CAR-engineered NK cells combining innate and adaptive antitumor mechanisms	EGFR, HER2, Mesothelin and other tumor antigens	Reduced toxicity, lower risk of CRS, allogeneic applicability, off-the-shelf potential	Limited persistence, reduced expansion, microenvironment suppression	CAR-NK investigational platforms	(4, 40, 41, 80)
Tumor-Infiltrating Lymphocyte (TIL) Therapy	Expansion and reinfusion of naturally occurring tumor-reactive lymphocytes	Multiple endogenous tumor antigens and neoantigens	Broad antigen recognition, reduced antigen escape	Complex manufacturing, variable expansion efficiency, limited persistence	Autologous TIL therapies	(42, 43, 46)
TCR-Engineered T Cell Therapy	Genetically modified T cells expressing tumor-specific TCRs	Intracellular neoantigens and tumor-associated peptides	Ability to target intracellular antigens, highly personalized therapy	HLA restriction, antigen presentation loss, immune suppression	Neoantigen-specific TCR-T therapies	(44–46, 62)

While the therapeutic use of adoptive cellular immunotherapy has revolutionized treatment for several hematologic malignancies, translating this success into NSCLC may be a much greater challenge. Solids tumors, in contrast to hematologic cancers, have several biological impediments: antigen heterogeneity, restricted trafficking of tumor-infiltrating lymphocytes, T-cell exhaustion, complex stromal architecture and strong suppressor TME. Furthermore, the complexity of the manufacturing process, toxicities associated with the treatment, and the high production costs still limit more widespread use of the treatment in the clinic. These limitations may be addressed by future improvements in immune engineering, gene editing, programmable cellular therapies and rational combinations, and will help clarify the potential for adoptive cellular therapy (ACT) to deliver durable clinical responses in patients with NSCLC. As a clinical approach, ACT is still largely experimental in the context of NSCLC; however, tailor-made clinical trials are likely to be the most suitable setting for the use of ACT in carefully selected patients. Treatment of patients with molecularly defined tumors that are expressing target antigens that are appropriate for the tumor and have suitable immune characteristics that will allow combination with, for example, immune checkpoint blockade or other immune-modulating therapies that will overcome the immunosuppressive TME is likely to be the focus for future clinical application. In order to bring the drug into the wider market, its efficacy must be proven over a long period of time, its production scaled up, and toxicity and cost of therapy minimized (48).

## 6 TME and immune resistance in NSCLC

### 6.1 Immunosuppressive components of the NSCLC microenvironment

**Figure 2:**
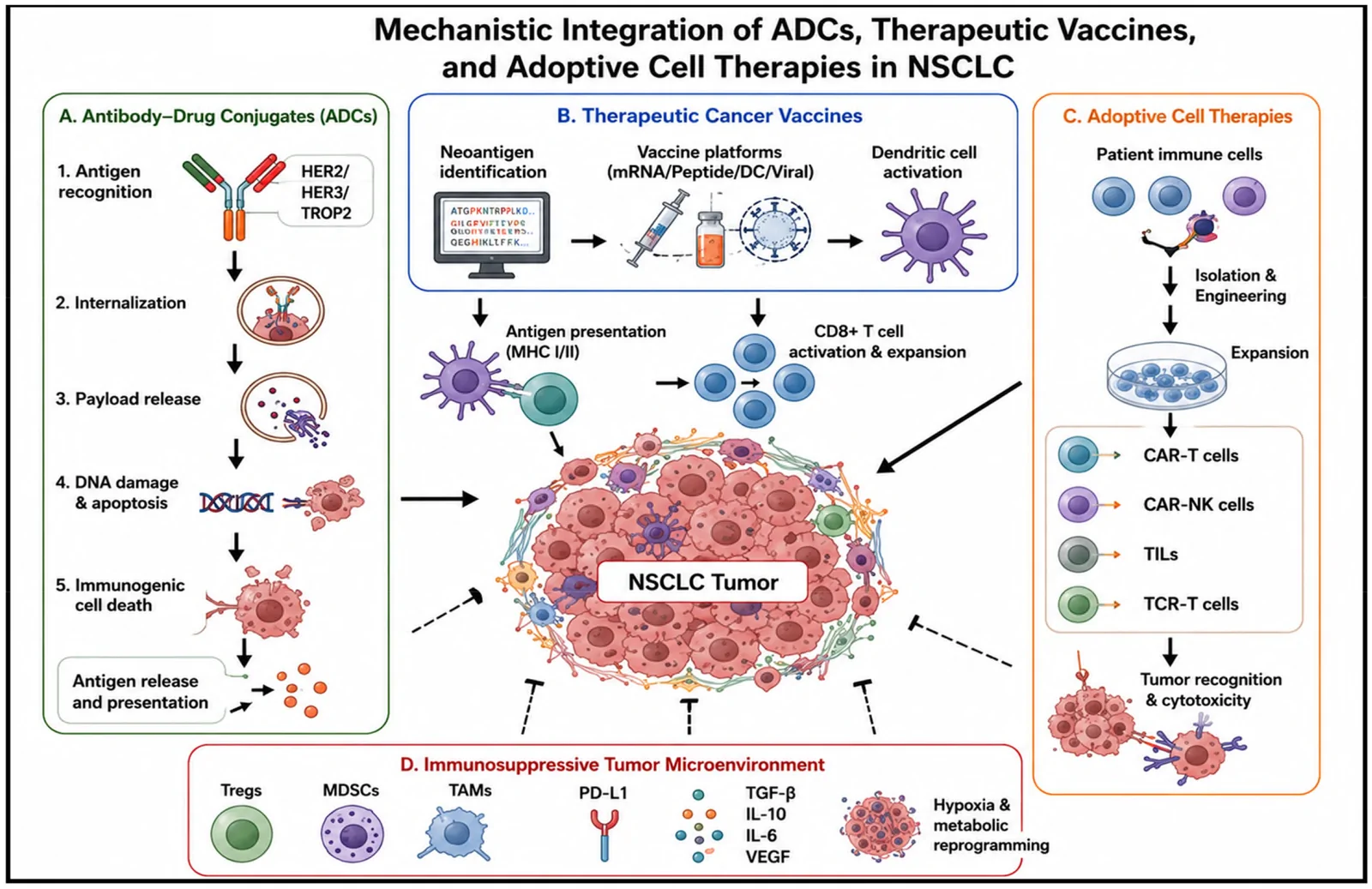
Mechanistic combination of ADCs, therapeutic cancer vaccines, and adoptive cell therapy in NSCLC. This schematic illustrates the complementary mechanisms that link to antitumor activity achieved by ADCs, therapeutic cancer vaccines, and adoptive cellular therapy in the context of NSCLC. Selectivity is achieved by binding tumor-associated antigen targets like HER2, HER3, TROP2, and their internalization, inducing tumor cell apoptosis and creating immunogenic tumor cell death for release of tumor antigens and presentation. Therapeutic cancer vaccines are based on neoantigen identification and the use of various vaccine platforms, such as mRNA, peptide, dendritic cell, and viral vector vaccines, which are used to promote antigen presentation, dendritic cell activation, and to enhance tumor-specific CD8+ T cell responses. Adoptive cell therapies rely on the extraction, modification, and proliferation of a patient’s own immune cells, such as CAR-T cells, CAR-NK cells, T-cell receptor (TCR)-engineered T cells, and TILs that can target and kill specific types of cancer. In addition, the Treg and MDSCs in the TME, TAMs, immune checkpoints, immunosuppressive cytokines, hypoxia, and metabolic reprogramming are highlighted and may affect therapeutic efficacy and lead to immune resistance.

The TME of NSCLC is an extremely dynamic and immunologically complex ecosystem that consists of various malignant cells, stromal components, immune cells, extracellular matrix proteins, cytokines, and vascular networks. Immunotherapeutic approaches, such as ADC, therapeutic vaccines, and adoptive cell therapies, focus on inducing antitumor immunity, but may be limited by the immunosuppressive properties of the NSCLC microenvironment. One of the prominent immunosuppressive immune populations in NSCLC tumors is Tregs. These cells allow the growth of tumors by secreting inhibitory cytokines, including transforming growth factor-β (TGF-β) and interleukin-10 (IL-10) (50), thereby delivering immune tolerance. Furthermore, MDSCs are strong players in immune dysfunction in the case of NSCLC, which curtails T-cell proliferation, hampers the presentation of antigens, and creates a tumor-associated TME. These cells are built up in response to chronic inflammatory stimuli and suppress the immune system against tumors via arginase activity and via production of inhibitory cytokines as well as generation of reactive oxygen species. TAMs, especially with M2 polarized macrophages, promote the progression of tumors by stimulating neo-angiogenesis, tumor dissemination, tissue remodeling, and immune dysregulation. Other cytokine pathways also contribute to the immunosuppressive TME of NSCLC. Cytokines such as IL-6, IL-10, vascular endothelial growth factor (VEGF), and TGF-β impair immune cell function, decrease antigen presentation, and contribute to resistance to immunotherapies. The combination of these immunosuppressive effects creates a potent microenvironment that hinders the success of next-generation precision immuno-oncology approaches in NSCLC (51). The mechanistic interplay between ADCs, therapeutic cancer vaccines, adoptive cellular therapies, and the immunosuppressive TME in NSCLC is illustrated in **Figure 2**.

### 6.2 Immune escape and therapeutic resistance

In NSCLC, mechanisms of immune escape play an important role in treatment resistance and are critical determinants of the clinical success of current immunotherapeutic strategies. Under selective pressure from both the immune system and therapeutic agents, tumor cells constantly evolve in ways that allow them to evade immune recognition and resist the effects of systemic therapy. The ability to downregulate or lose antigen expression is one of the major mechanisms of immune escape used by tumors targeted by vaccines, ADCs, or adoptive cellular therapies (52). In addition, NSCLC tumors are often characterized by antigenic heterogeneity, which can further compromise treatment responses and allow resistant tumor cell populations to survive. Other important mechanisms of resistance include the upregulation of immune checkpoint molecules, including PD-L1, cytotoxic T-lymphocyte-associated protein-4 (CTLA-4), lymphocyte activation gene-3 (LAG-3), and T-cell immunoglobulin and mucin-domain-containing protein-3 (TIM-3). These inhibitory pathways suppress T-cell activation and contribute to T-cell exhaustion within the TME. In addition to playing a central role in immune resistance, metabolic reprogramming is also a critical determinant of immune dysfunction. NSCLC tumors are associated with metabolic changes like increased glycolysis, hypoxia, lactate production, and competition for nutrients that negatively affect immune cell survival and function. A lack of glucose, amino acids, and oxygen in the tumor prevents the activity of cytotoxic lymphocytes and promotes the growth of immunosuppressive cell subsets. These adaptive resistance mechanisms collectively decrease the efficacy and durability of immunotherapies in NSCLC (53).

### 6.3 Strategies to remodel the TME

Extensive research is currently underway to find strategies that can remodel the NSCLC microenvironment to increase the response to immunotherapy as the TME is believed to play a key role in therapeutic resistance. Combination immunotherapy, which involves combating immune suppression and boosting antitumor immunity, is one of the most promising methods so far. The combination of therapeutic vaccines, adoptive cell therapies, ICIs, and ADCs provides a chance to stimulate immune responses, bypass immune cell exhaustion, and boost the amount of tumor antigens being delivered. The combination of these approaches can be used to achieve therapeutic synergy and to bypass resistance associated pathways (54). In addition, cytokine modulation is another important approach to reprogramming the tumor environment. A balance between immunosuppressive cytokines (TGF-β and IL-10) and immunostimulatory cytokines (IL-2, IL-12, and interferons) has been demonstrated to be crucial in restoring immune cell functions and inducing powerful antitumor immune responses. Furthermore, stromal targeting is under investigation to increase the infiltration of immune cells and delivery of therapeutic agents to solid tumors. NSCLC tumors have a complex stroma and matrix, which form physical barriers to infiltration by immune cells and to drug delivery. Normalizing the tumor blood vessels, degrading elements of the extracellular matrix, and targeting cancer-associated fibroblasts could facilitate the delivery and efficacy of immunotherapeutic agents (55). Together, these approaches to TME remodeling are anticipated to significantly improve the clinical outcomes of future precision immuno-oncology therapies for NSCLC.

TME is still one of the major factors to impact the success or failure of therapy across all precision immuno-oncology platforms in NSCLC. Interestingly, new research has emerged that shows that modulation of TME is likely not a therapeutic modality but rather is critical to maximize the clinical potential of therapeutic cancer vaccines, adoptive cellular immunotherapies, and ADCs. Thus, future treatment paradigms are anticipated to combine TME remodeling approaches with combination therapies based on biomarkers to overcome immune resistance, optimize patient selection, and develop more durable clinical responses (54, 55).

## 7 Biomarkers and precision patient stratification

### 7.1 Predictive biomarkers in NSCLC immunotherapy

The development of predictive biomarkers has become increasingly crucial for ensuring appropriate patient selection and optimal therapeutic outcomes in the field of NSCLC immunotherapy. Currently, expression of PD-L1 is one of the most commonly used biomarkers to guide ICI therapy. Tumors or immune cells with high levels of PD-L1 tend to be more responsive to PD-1/PD-L1 blockade, but there is considerable heterogeneity among patients. While clinically useful, PD-L1 expression alone is not sufficient to accurately predict the duration of therapeutic response because tumors are heterogeneous and exhibit dynamic biological behavior (56). Another significant biomarker related to the number of somatic mutations in the tumor genome is tumor mutational burden (TMB). A high TMB can drive neoantigen generation, which is believed to enhance tumor immunogenicity and sensitivity to immunotherapy (57). Another important emerging liquid biopsy technique that has garnered significant interest is ctDNA analysis, which can track tumor evolution, treatment response, and the acquisition of resistance mutations throughout the course of therapy. In addition, neoantigen burden has emerged as a key biomarker in precision immuno-oncology, particularly in the development of therapeutic vaccines and adoptive cellular therapies. Personalized immunotherapies may exhibit greater antitumor activity in tumors with a higher neoantigen burden (58).

### 7.2 Biomarkers for ADC response

For ADCs in NSCLC, clinical efficacy is closely linked to appropriate patient stratification based on biomarkers. Antigen expression profiling is a key component of therapeutic eligibility and is used to predict ADC responsiveness. Generally, TAAs that are highly expressed show a strong correlation with ADC binding, internalization, and therapeutic efficacy, including HER2, TROP2, HER3, and MET. The relevance of HER2 mutation status and protein overexpression has increased as a biomarker for HER2-directed ADCs in NSCLC (59). Likewise, TROP2 expression is being studied as a predictive biomarker for ADCs targeting TROP2. However, biomarker interpretation in ADC therapy remains challenging because of intratumoral heterogeneity, dynamic antigen expression, and variations in assay standardization. Patients with low levels of antigen expression may still benefit from the bystander killing effects exhibited by certain ADCs. Comprehensive molecular and antigen characterization will therefore become increasingly important in guiding optimal precision medicine approaches for ADC therapy in NSCLC (60).

### 7.3 Biomarkers for cellular and vaccine therapies

The development of biomarkers is also crucial for enhancing the efficacy and personalization of therapeutic vaccines and adoptive cell therapies in NSCLC. Immune infiltration signatures characterized by high densities and activity of immune cells, such as tumor-infiltrating cytotoxic T cells and dendritic cells, can provide valuable information regarding responsiveness to immunotherapies. Tumors that are already infiltrated with immune cells tend to exhibit greater responses to immunotherapeutic treatments, whereas tumors with minimal immune activation generally demonstrate poorer responses (61). Another promising biomarker-based approach involves analysis of the T-cell receptor (TCR) repertoire, which can assess immune diversity, clonality, and antigen-specific immune responses. An increased number of TCR clonotypes may indicate effective immune activation and tumor recognition following vaccination or adoptive cell therapy (ACT). Moreover, neoantigen profiling, cytokine signatures, HLA typing, and immune checkpoint expression are additional promising biomarkers currently being investigated for personalized immunotherapy selection. The longitudinal assessment of these biomarkers may improve patient stratification and clinical outcomes in NSCLC patients undergoing precision immuno-oncology therapies (62).

### 7.4 AI and multi-omics in precision immuno-oncology

AI and multi-omics technologies are revolutionizing the discovery of biomarkers and therapeutic personalization in NSCLC. Using AI and computational methods, we can start to explain complex relationships between genomic, transcriptomic, proteomic, radiomic, and clinical variables in order to discover predictive biomarkers related to therapeutic response, resistance, and patient survival. Algorithms using machine learning are being applied to predict neoantigen immunogenicity, optimize vaccine design, open new targets for ADC, and stratify patients for personalized immunotherapeutic approaches. The combination of genomics, transcriptomics, proteomics, metabolomics, and single-cell sequencing technologies allows for more detailed understanding of NSCLC tumor biology and interactions with the immune system (63). Multi-omics profiling can be used for discovery of molecular signatures linked to immune activation, tumor heterogeneity, metabolic adaptation, and resistance mechanisms. Furthermore, the ability to integrate multi-omics datasets using AI could help create highly personalized precision immuno-oncology approaches that improve their therapeutic efficacy and aid in clinical decision making. In the future, the application of AI for biomarker discovery and systems-level tumor profiling will be increasingly important in the management of NSCLC (64). The principal biomarkers currently supporting patient stratification, therapeutic selection, treatment monitoring, and precision immuno-oncology approaches in NSCLC are summarized in **Table 3**.

**Table 3:** Biomarkers supporting precision immuno-oncology in NSCLC.

Biomarker category	Biomarker	Biological significance	Clinical utility	Therapeutic relevance	References
Immune Checkpoint Biomarkers	PD-L1 Expression	Reflects immune inhibitory signaling within tumors	Patient selection for PD-1/PD-L1 inhibitors	Predictive biomarker for checkpoint blockade	(8, 54, 55, 61, 70, 74, 87)
Genomic Biomarkers	Tumor Mutational Burden (TMB)	Represents overall mutational load and neoantigen generation	Predicts likelihood of immunotherapy response	Relevant for vaccines, ICIs, and cellular therapies	(57, 61, 67, 74, 78)
Liquid Biopsy Biomarkers	Circulating Tumor DNA (ctDNA)	Monitors tumor evolution and treatment response	Dynamic disease monitoring and resistance detection	Precision treatment adaptation	(58, 61, 63, 64)
Neoantigen Biomarkers	Neoantigen Burden	Reflects availability of tumor-specific immune targets	Candidate selection for personalized vaccines and TCR therapies	Precision immunotherapy development	(30, 33, 67, 78, 81)
ADC Biomarkers	HER2 Expression/Mutation	Determines target availability for HER2-directed ADCs	Patient selection	Trastuzumab deruxtecan eligibility	(16, 18, 59, 75)
	HER3 Expression	Associated with ADC internalization and efficacy	Patient stratification	Patritumab deruxtecan	(17, 19, 59, 76)
	TROP2 Expression	Widely expressed NSCLC target	Predictive biomarker for TROP2 ADCs	Datopotamab deruxtecan	(18, 59, 60)
Immune Profiling Biomarkers	Tumor Immune Infiltration Signature	Reflects abundance of cytotoxic immune cells	Predicts responsiveness to immunotherapy	Cellular therapy and vaccine selection	(50, 51, 54, 56, 74)
Cellular Therapy Biomarkers	TCR Repertoire Diversity	Indicates immune clonality and antigen-specific responses	Monitoring adoptive cell therapy outcomes	Personalized cellular therapies	(45, 46, 62, 74)

Patient stratification is key for the development of precision immuno-oncology, more and more, rather than therapeutic innovation. While some biomarkers, like the expression of PD-L1 and tumor mutational burden, have aided with patient selection, no single marker has proven to be sufficiently accurate when used alone due to tumor heterogeneity, the variability of the TME, and temporal changes in biomarker expression. Such restrictions are among the principal factors that may contribute to the variety of clinical outcomes in patients with seemingly identical molecular features after immunotherapy. Precision oncology in the future is expected to depend on the ability of genomic, transcriptomic, proteomic, radiomic, and immune profiling to be integrated with patient treatment recommendations from AI and biomarker analysis to inform personalized therapeutic decision making. In addition to informing therapeutic responses, these multidimensional biomarker platforms will need to be used to choose the right immunotherapeutic modality, to determine which patient will gain the greatest benefit from single or multi-agent therapies, and to enable the adoption of precision immuno-oncology in everyday clinical care. These biomarker-driven approaches are likely to aid in treatment and therapy selection, ensure optimal combination treatments, and ultimately yield better clinical outcomes for next generation precision immuno-oncology in NSCLC (61).

## 8 Combinational and integrative therapeutic strategies

### 8.1 ADCs combined with ICIs

Combinational therapy with ADC and ICIs is one of the most promising combinational approaches in precision immuno-oncology for the treatment of NSCLC. Besides directly killing tumor cells, ADCs can also enhance tumor immunogenicity by inducing immunogenic tumor cell death, releasing TAAs, and altering the TME. These effects can also boost the activation of dendritic cells and antigen presentation to T cells, making them more responsive to checkpoint blockade drugs targeting the PD-1/PD-L1 and/or CTLA-4 pathways. At the same time, ICIs revitalize exhausted T-cell functions and improve adaptive antitumor immune responses, potentially resulting in a synergistic therapeutic effect (65). Multiple clinical trials are underway for combinations with ADCs in NSCLC, including the combination of an ADC targeting HER2, HER3, and TROP2. Currently, these combinations seem to be a positive treatment option for objective response rates and PFS in a subset of patient populations, especially those with tumors that are resistant to conventional immunotherapy. Toxicity management is important, however, because combination therapy can provide for a higher risk for overlapping pulmonary and immune related adverse events (irAEs) like pneumonitis and interstitial lung disease. Therefore, choosing patients based on biomarkers and optimizing dosing regimens is critical for maximizing therapeutic benefit with the least toxic side effects from treatment (66).

### 8.2 Vaccine-based combination strategies

Cancer vaccines have proven to be of limited value when used as stand-alone therapy and have increasingly been used in combination with other systemic therapy to boost immune activation. A novel approach is to use therapeutic vaccines in combination with ICIs. Vaccines influence antigen-specific priming and expansion of T cells and checkpoint blockade enables the restoration of immune effector function in the TME by eliminating T-cell exhaustion. This complementary interaction can have a significant impact on the ability of the antitumor immune response to become strong and durable in NSCLC. Therapeutic vaccines are also being explored in combination with other agents, such as ICIs, chemotherapy, radiotherapy, targeted drugs, and ADCs (67). Conventional therapies can cause immunogenic tumor cell death with the consequent increased release of tumor antigens and a corresponding increase in vaccine-mediated immune activation. Radiotherapy can further promote immune cell infiltration into tumors and increase cytokine release within the TME, thereby helping to convert immunologically “cold” tumors into more immune-responsive “hot” tumors. Preclinical studies of personalized neoantigen vaccines combined with PD-1 inhibitors have shown promising results with respect to immunogenicity and clinical activity. These combination strategies are anticipated to significantly enhance therapeutic efficacy and broaden the clinical applicability of cancer vaccines in NSCLC (68).

### 8.3 Cellular therapies combined with targeted therapy

Another promising approach for overcoming resistance mechanisms in NSCLC is the combination of adoptive cellular therapies with targeted therapies. Targeted therapies directed against oncogenic drivers such as EGFR, ALK, and Kras may alter tumor signaling pathways in ways that enhance immune responsiveness and modify the TME. The combination of targeted agents with CAR-T cells, CAR-NK cells, or TCR-engineered T cells may enhance tumor antigen expression, increase immune cell infiltration, and reduce immunosuppressive signaling within tumor tissues (69). Targeted therapies may also sensitize tumor cells to immune-mediated killing by altering apoptotic pathways and tumor metabolism. EGFR inhibition, for instance, has been associated with changes in PD-L1 expression and immune modulation that may improve the effectiveness of adoptive cellular therapies. Likewise, anti-VEGF therapies may promote vascular normalization within tumors and facilitate immune cell infiltration. Although promising interactions have been observed, several factors must be considered during the development of these combination strategies, including overlapping toxicities, dynamic tumor adaptation, and antigen heterogeneity (70).

### 8.4 Personalized combination immunotherapy paradigms

The growing complexity of NSCLC biology has emphasized the need for individualized combination immunotherapy approaches based on the molecular and immune characteristics of each patient. Advances in high-precision genomic profiling, biomarker discovery, and immune monitoring have paved the way for the development of precision-guided therapeutic strategies that combine multiple immunotherapeutic modalities according to tumor-specific characteristics. Such strategies may incorporate ADCs, therapeutic vaccines, adoptive cellular therapies, ICIs, and targeted therapies based on antigen expression levels, tumor mutational burden, neoantigen load, and immune infiltration status (71). These personalized strategies have the potential to enhance therapeutic efficacy by simultaneously targeting multiple resistance mechanisms while minimizing opportunities for tumor escape. In addition, personalized treatment paradigms supported by real-time monitoring of molecular tumor profiles through circulating tumor DNA and immune profiling may enable adaptive treatment modifications throughout the course of disease progression. The integration of AI and multi-omics analyses is also being increasingly explored for developing predictive algorithms capable of optimizing immunotherapy combinations for individual patients with NSCLC (72).

### 8.5 Rationale for multimodal precision immuno-oncology

The rationale behind multimodal precision immuno-oncology in NSCLC is based on the recognition that no single therapeutic approach is sufficient to address the biological complexity and heterogeneity of advanced lung cancer. Mechanisms such as tumor evolution, immune suppression, genomic instability, and adaptive resistance collectively contribute to therapeutic failure when treatment modalities are used in isolation. The integration of ADCs, therapeutic vaccines, and adoptive cellular therapies provides opportunities for multi-level targeting of tumor cells through complementary mechanisms involving direct cytotoxicity, immune activation, antigen-specific targeting, and modulation of the TME (73). Through the induction of immunogenic tumor cell death and tumor antigen release, ADCs can enhance vaccine priming and improve the effectiveness of cellular therapies. Therapeutic vaccines may increase the pool of tumor-reactive T cells that can subsequently be leveraged by adoptive cellular therapies and immune checkpoint blockade. Likewise, engineered immune cells can eliminate resistant tumor populations that escape targeted or vaccine-mediated killing. This multimodal therapeutic approach closely aligns with the principles of precision medicine by enabling personalized treatment strategies based on the molecular and immunological characteristics of individual patients. Several existing integrative precision immuno-oncology approaches have the potential to substantially improve response durability, reduce therapeutic resistance, and facilitate the development of more effective systemic treatments for patients with NSCLC (74). The major combinational approaches are currently being investigated in NSCLC, together with their scientific rationale, therapeutic advantages, and associated challenges, are summarized in **Table 4**.

**Table 4:** Emerging combination strategies in precision immuno-oncology for NSCLC.

Combination strategy	Scientific rationale	Potential benefits	Current challenges	References
ADC + Immune Checkpoint Inhibitor	ADC-induced immunogenic cell death enhances antigen presentation and T-cell activation	Improved response rates, enhanced immune activation, delayed resistance	Pneumonitis, overlapping toxicity, biomarker optimization	(55, 59, 65, 66, 75)
Therapeutic Vaccine + PD-1/PD-L1 Blockade	Vaccine primes tumor-specific T cells while checkpoint inhibitors reverse T-cell exhaustion	Durable immune responses, enhanced T-cell expansion	Immune-related adverse events, patient heterogeneity	(25, 29, 31, 67, 77)
Therapeutic Vaccine + Radiotherapy	Radiation promotes antigen release and inflammatory signaling	Improved vaccine immunogenicity and immune infiltration	Optimal scheduling and dosing	(29, 67, 68, 77, 87)
ADC + Therapeutic Vaccine	ADC-mediated tumor destruction increases antigen availability for vaccine-induced immunity	Enhanced immune priming and broader tumor targeting	Limited clinical data	(59, 65–67)
CAR-T/CAR-NK + Targeted Therapy	Targeted therapies modulate tumor signaling and improve immune susceptibility	Improved tumor infiltration and immune-mediated killing	Antigen heterogeneity and resistance	(35, 39, 40, 44, 79, 80)
Cellular Therapy + Checkpoint Blockade	Reversal of T-cell exhaustion improves cellular therapy persistence	Enhanced durability and antitumor efficacy	Increased immune toxicity risk	(35, 47, 48, 79, 87)
Multimodal Precision Immunotherapy (ADC + Vaccine + Cellular Therapy ± ICI)	Simultaneous targeting of tumor cells, immune activation, and resistance pathways	Comprehensive antitumor activity, personalized treatment paradigms	Manufacturing complexity, cost, toxicity management	(20, 65, 67, 81, 87, 88)

Altogether, the available data suggests that none of the existing immunotherapies are likely to be a panacea against the biological intricacies of NSCLC in isolation. From clinical experience, it became clear that therapeutic failure often occurs when multiple mechanisms of tumor heterogeneity, antigen escape, immune suppression, and adaptive resistance are present at the same time and cannot be effectively treated with one therapeutic modality. Such observations have spurred the current research that directs efforts away from single therapeutic modalities and into rational multimodal strategies that simultaneously kill the tumor cells, overcome immune system suppression, and take advantage of complementary mechanisms of immune activation. Overall, new treatment concepts in advanced NSCLC will not involve sequential use of individual immunotherapies. Rather, precision systemic therapy will be based on multimodal treatment strategies designed by tumor biology, molecular alterations, immune phenotype, and prior treatment exposures using a biomarker-guided approach. Combining these different therapeutic approaches with biomarker optimization could therefore be argued to be the most valuable treatment in the future, with adaptive clinical trial designs, molecular profiling, and long-term monitoring of biomarkers, providing the promise of maximizing treatment sequences and combinations for individual patients (63, 64).

## 9 Current clinical trials and translational landscape

### 9.1 Ongoing clinical trials in ADCs

The clinical advancement of ADCs in NSCLC has made remarkable progress, with several ADCs currently under investigation in numerous molecularly targeted subsets of NSCLC. The clinical efficacy of HER2-directed ADCs in NSCLC patients with HER2 mutations has been striking and has paved the way for further ADC development in thoracic malignancies. Advanced and treatment-refractory NSCLC are areas where TROP2-targeted ADCs are currently under development, including in combination with ICIs (75). Also, HER3-directed ADCs such as patritumab deruxtecan are showing promising activity in TKI-resistant tumors with EGFR mutations. New clinical trials are targeting patient stratification, combination therapy, and toxicity management using biomarkers. Current studies are focused on mechanisms of resistance, predictive biomarkers, and novel engineering strategies like bispecific and dual-payload ADCs, in addition to clinical efficacy. These clinical advances are likely to expand the role of ADCs in the precision treatment of NSCLC (76).

### 9.2 Clinical development of cancer vaccines

Therapeutic cancer vaccines are progressing through both early- and late-phase clinical trial evaluations in NSCLC. The major platforms being explored include mRNA vaccines, dendritic cell vaccines, viral vector vaccines, and personalized neoantigen vaccines. To overcome tumor-induced immune suppression, several vaccine strategies are being investigated in combination with PD-1/PD-L1 inhibitors to enhance immune activation. Early clinical studies have demonstrated promising safety profiles, immunogenicity, and expansion of tumor-specific T-cell populations (77). Significant progress has been made in the field of personalized mRNA vaccines, which are gaining considerable attention due to advances in next-generation genomic sequencing and computational neoantigen prediction. These customized vaccine platforms enable the targeting of patient-specific mutations and may improve therapeutic effectiveness. However, further clinical trials are needed to define long-term efficacy, optimal antigen selection strategies, and the role of vaccines in different therapeutic settings of NSCLC, including the adjuvant and metastatic settings (78). Despite encouraging immunogenicity, multiple randomized clinical trials of therapeutic cancer vaccines have failed to demonstrate consistent improvements in overall survival when administered as monotherapy. These disappointing clinical outcomes have highlighted the limitations of non-personalized vaccine strategies and have shifted current research toward individualized neoantigen vaccines integrated with immune checkpoint blockade and biomarker-guided patient selection.

### 9.3 Emerging cellular therapy trials in NSCLC

The clinical evaluation of adoptive cellular therapies in NSCLC is progressing rapidly, although most approaches remain in the early stages of development. CAR-T cell therapies are currently being evaluated in phase I and phase II clinical trials against targets such as EGFR, mesothelin, HER2, MUC1, and other TAAs (79). Likewise, CAR-NK therapies have attracted significant interest because of their favorable safety profile and potential as allogeneic “off-the-shelf” therapeutic products. Other strategies under investigation include TCR-engineered T-cell therapies and TIL therapies designed to target neoantigen-specific immune responses in NSCLC. While there is promising progress observed in pre-clinical research and in early clinical trials, there are significant hurdles to consider such as persistence, trafficking, and resistance of immune cells in the solid TME (80). New clinical trials are, therefore, adopting advanced engineering technologies, cytokine support strategies, and combination therapy involving ICIs for enhanced therapeutic efficacy. The results of these current clinical trials will likely have major impacts on how cellular immunotherapies are applied to the treatment of NSCLC in the future. Early clinical studies have shown feasibility and clinical safety; however, the durability of clinical responses is not as good as for hematological malignancies. Today, however, clinical development is increasingly orienting to the development of new immunotherapeutic approaches to combat resistance mechanisms specific to solid tumors, the selection of the right patients based on better markers, and the use of rational combinations, rather than on further advances in cellular engineering.

### 9.4 Translational challenges

Next-generation precision immune-oncology drugs have shown positive outcomes, but there are some translatable hurdles to clear before these therapeutic platforms become broadly used in NSCLC treatment. Complexity for manufacturing is a significant issue, especially for individualized cellular therapies and neoantigen vaccines, where each batch must be manufactured in a different way, and specialized manufacturing facilities are of great importance with extensive quality control measures. These manufacturing requirements significantly increase the cost of treating products and restrict access to treatment in many health care environments. The complexity of biologic products, advanced engineered cell products, and combination therapies also challenges the regulatory system (81). To achieve successful clinical translation, it is crucial to standardize manufacturing processes, set up robust biomarker validation systems, and ensure comprehensive long-term safety monitoring programs. Toxicity control, especially cytokine release syndrome, neurotoxicity, and pulmonary toxicities with immune therapies are also critical factors. Furthermore, patient heterogeneity and the changing response to therapy and tumor dynamics pose significant challenges for clinical decision making and therapy optimization. There are several barriers that remain to be overcome for the successful translation of precision immuno-oncology strategies into clinical care for NSCLC patients (82).

While clinical development for the various types of ADCs, therapeutic cancer vaccines, and adoptive cellular therapies is progressing quickly, these platforms are at varying clinical maturity. While therapeutic cancer vaccines and adoptive cellular therapies still have significant biological, manufacturing, and regulatory hurdles to overcome to be broadly clinically available, ADC has already shown clinical benefit in biomarker-selected patients and are increasingly being rolled into the clinic. Going forward, robust biomarker validation, innovative adaptive clinical trial designs, scalable manufacturing technologies, and multidisciplinary collaboration to enable the successful clinical translation of next-generation precision immuno-oncology strategies for NSCLC will be required. Overall, this clinical status suggests that the use of ADC products is developing into clinical practice, while therapeutic cancer vaccines and adoptive cellular therapies are still mostly in the investigational stage. As a result, therapeutic expectations, biomarker selection in patient dosing/trials, and future precision therapeutic algorithms should be informed by these differences in NSCLC (44).

## 10 Future perspectives and emerging innovations

**Figure 3:**
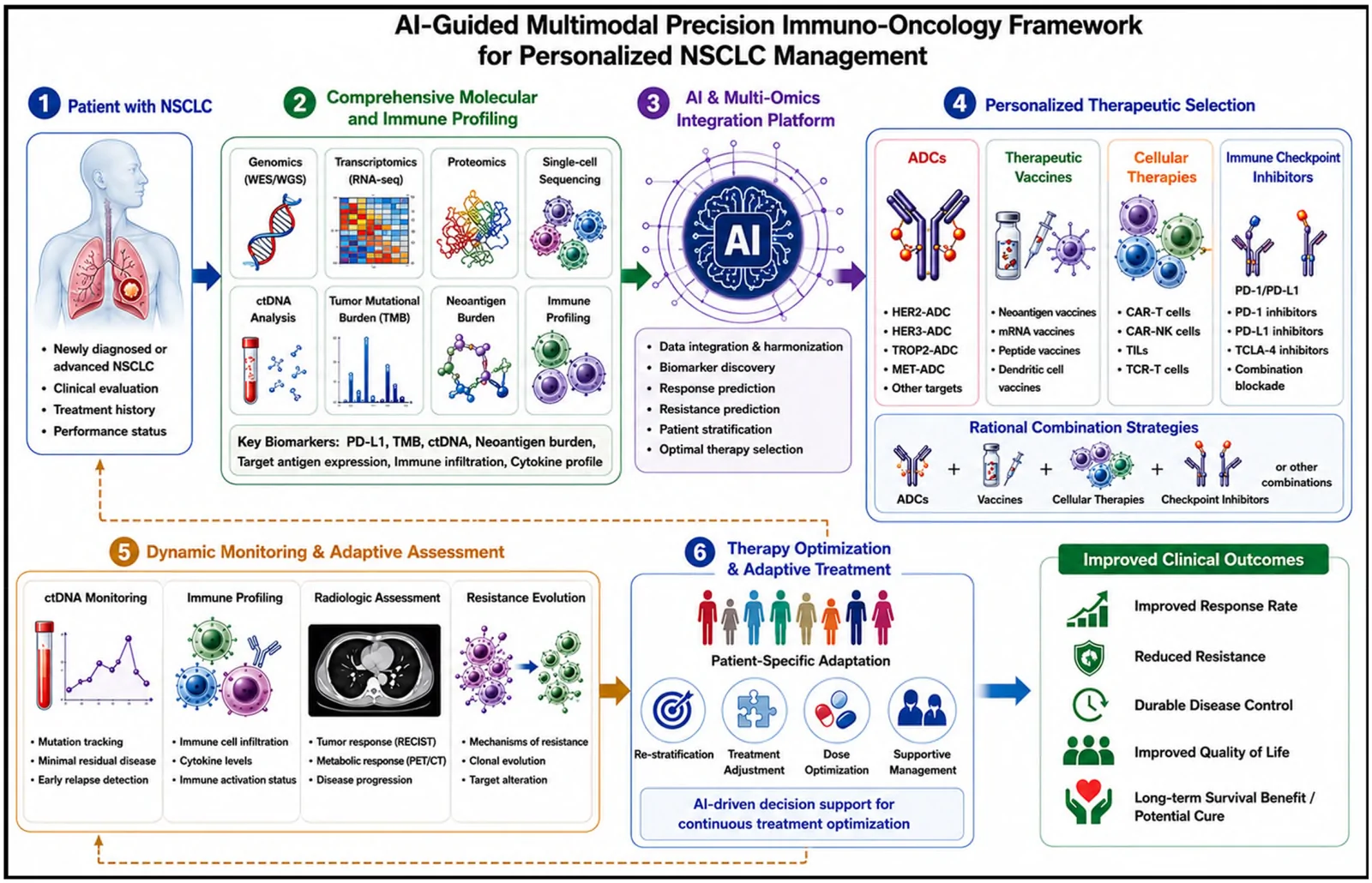
Multimodal precision immuno-oncology approach for personalized care of NSCLC enabled by AI. This figure shows a conceptual framework for the implementation of AI and multi-omics technologies for personalized precision immuno-oncology in NSCLC. This process starts with large-scale molecular and immune characterization by using genomic, transcriptomic, proteomic, single-cell sequencing, ctDNA, tumor mutational burden (TMB), and neo-antigen burden data, in addition to immune characterization data. These data can be combined in AI-driven analytical platforms to detect predictive biomarkers, stratify patients, predict the response to the therapy, and support optimization of therapy choice. Individual therapeutic strategies may involve ADCs, therapeutic cancer vaccines, adoptive cellular therapies, ICIs, or rational multimodal combinations, based on these analyses. Assessing ctDNA, immune markers, and resistance markers as well as performing radiological evaluation allows for optimizing treatment throughout the course of the disease. In the end, this precision-guided framework may increase response rates, overcome therapeutic resistance, provide sustained disease control, improve quality of life, and improve long-term survival for patients with NSCLC.

The future of NSCLC treatment is shifting towards highly personalized treatment modalities based on molecular profiling, immune characterization, and the selection of therapeutic options guided by biomarkers. The adoption of novel technological advancements in genomic sequencing, neo-antigen prediction, and liquid biopsy is enabling the development of individualized therapeutic approaches for every patient and cancer. Combination therapies, including new personalized treatment paradigms that integrate ADCs, therapeutic vaccines, and adoptive cellular therapies, may significantly improve the precision and lasting therapeutic response, and minimize the toxicity of these therapies (83). AI is revolutionizing the creation of next-generation immune therapeutics in NSCLC at a rapid pace. Utilization of machine learning and computational modelling approaches to identify novel therapeutic targets, create ADC designs, predict neo-antigen immunogenicity, and patient stratification strategies are underway. There is a potential for the use of AI in precision oncology to assess large datasets of clinical, genomic, and imaging information to inform treatment decisions and real-time patient monitoring, adding another layer of analytical power (84). An AI-guided multimodal precision immuno-oncology framework integrating molecular profiling, biomarker discovery, personalized therapeutic selection, adaptive monitoring, and treatment optimization in NSCLC is illustrated in **Figure 3**.

Newly available programmable and synthetic immunotherapies offer fresh opportunities for therapeutic specificity and controllability of NSCLC. Synthetic biology approaches are driving the design and engineering of logic-gated CAR-T cells, programmed immune circuits, inducible cytokine systems, and tumor-responsive therapeutic platforms that can be selectively activated within tumors. These new technologies could improve the accuracy of the treatment, reduce systemic toxicity, and off-target immune stimulation (85). Recent advances in gene editing, engineering of immune cells, and biomaterial delivery are producing a significant boost in the potential of cellular immunotherapy. Improving immune persistence and resistance to immune suppression and increasing antineoplastic efficacy are pursued by developing novel genome editing approaches with CRISPR/Cas, armored CARs, multi-target immune cells, and cytokine-engineered therapies. Additionally, next-generation delivery technologies might enable delivery and localization of therapeutic agents within intratumoral areas of the NSCLC tumors (86). While outcomes for patients with metastatic NSCLC have been historically poor, precision immuno-oncology therapy offers a new opportunity to achieve long-term control or even a cure of the disease for some patients. The ideal selection of biomarkers, combination of multiple therapeutic modalities, and use of immune engineering technologies may transform NSCLC into a more controlled and individualized disease. Future translational research, study design of new generation systemic drugs, and collaboration among the different specialties will be key to realizing the full therapeutic potential of precision immuno-oncology for NSCLC (87). Despite substantial therapeutic progress, several biological, clinical, and translational challenges continue to influence the successful implementation of precision immuno-oncology in NSCLC. The major challenges and potential future solutions are summarized in **Table 5**.

**Table 5:** Major challenges and future solutions for next-generation precision immuno-oncology in NSCLC .

Challenge	Impact on therapy	Potential solutions	References
Tumor heterogeneity	Antigen escape and therapeutic resistance	Multi-target therapies, personalized treatment approaches	(18, 20, 50, 52, 71, 83)
Immunosuppressive TME	Reduced immune cell activity	Cytokine modulation, checkpoint inhibition, stromal targeting	(5, 50, 51, 55, 65)
T-cell exhaustion	Reduced cellular therapy efficacy	Armored CARs, checkpoint blockade combinations	(35, 47, 48, 87)
Limited biomarker accuracy	Inadequate patient selection	Multi-omics and AI-assisted biomarker discovery	(56–58, 61, 63, 64, 74, 89)
Manufacturing complexity	Restricted accessibility	Automated production platforms and allogeneic cell therapies	(36, 40, 48, 80, 82)
Treatment-associated toxicity	Limits therapeutic intensity	Precision dosing, programmable immune systems	(16, 22, 41, 79, 82)
High treatment costs	Reduced global accessibility	Scalable manufacturing and standardized platforms	(36, 40, 48, 66, 80)

The most important improvements in the coming decade will not be based on a single therapeutic platform but rather on the intelligent integration of therapeutic tools via biomarker-driven precision medicine, the AI-supported clinical decision-making process, and the adaptive multimodal use of immunotherapeutic approaches. To maximize therapeutic benefit, it is clear from current clinical experience that future success will require more than just the integration of increasingly sophisticated immunotherapies, but careful selection of patients, immune resistance, complementary therapeutic mechanisms, and application of rigorous clinical decision-making based on biomarkers. Further advancement of translational research, multi-disciplinary research collaboration and adaptive clinical trial design will therefore be crucial in delivering novel innovations to sustained clinical benefit in the treatment of patients with NSCLC (81, 87).

## 11 Conclusion

The field of NSCLC therapy has come a long way from the era of chemotherapy to precision-guided immuno-oncology therapies targeting tumor biology and immune dysregulation. A lot of effort has been put into using targeted therapeutics and ICIs, yet there is still a high frequency of treatment resistance and tumor heterogeneity, and the patients continue to escape treatment, limiting the long-term clinical success for a subset of patients. In this context, ADCs, therapeutic cancer vaccines, and adoptive cellular therapies are emerging as promising next-generation systemic therapeutic platforms with the potential to optimize tumor selectivity, enhance antitumor immune responses, and overcome resistance-associated mechanisms (88). The targeted delivery of cytotoxic agents through ADC, tumor-specific immune activation via therapeutic vaccines, and the immune-mediated tumor eradication via engineered cellular therapies are the means that enable these. In addition to these modalities and biomarker-driven precision medicine strategies, the management of NSCLC is quickly changing. Furthermore, the knowledge of the TME, resistance mechanisms, and multi-omics profiling is leading to the development of increasingly individualized and adaptive therapeutic approaches (89).

Regardless of impressive scientific progress, the three big precision immuno-oncology platforms (IOPs) mentioned in this manuscript are still at various stages of clinical maturity. Therapeutic cancer vaccines and adoptive cellular therapies have several key biological and clinical hurdles to overcome – immune tolerance, tumor heterogeneity, poor immune-cell trafficking, T-cell exhaustion, manufacturing complexity, and the highly immunosuppressive TME – which is a reason for why none of these therapies have been as successful in the clinic or as well established as ADCs, where validated molecular targets, improvements in linker chemistry, and patient selection based on biomarkers have paved the way for success. These observations suggest that not only will technological innovation continue to be essential for successful precision immuno-oncology, but also a greater understanding of tumor biology, rational patient selection based on biomarkers, and the ability to create integrated therapeutic strategies that can simultaneously neutralize multiple resistance pathways will be critical to the future of precision oncology. Continued clinical validation of therapeutic cancer vaccines and adoptive cellular therapies will likely be needed to establish these technologies in routine clinical use while ADCs are expected to remain a staple of routine clinical use as the technology matures.

Looking ahead in future, the next 5–10 years will see a shift from the development of single immunotherapeutic platforms to their intelligent integration into biomarker-guided precision oncology. More powerful biomarker validation, longitudinal multi-omics profiling, adaptive clinical trial designs, scalable manufacturing technologies, and rational combination of complementary immunotherapeutic modalities as potential means to overcome tumor heterogeneity and immune resistance should be investigated in future research. A single breakthrough therapy is unlikely to be the result of this; instead, continued successes in the clinic will most likely be achieved using multimodal treatment approaches that are designed and customized based on the molecular and immunological profile of the individual patient. Together, they could transform systemic treatment of NSCLC and accelerate the long-term benefits in patients, with durable, personalized and clinically meaningful long-term outcomes, towards precision immuno-oncology (81, 87–90).

## 12 Clinical significance7

- ADCs improve NSCLC treatment by selectively targeting tumors while reducing systemic toxicities.
- Therapeutic vaccines enhance tumor-specific immunity and support durable antitumor responses.
- Adoptive cell therapies offer new options for patients with resistant or refractory NSCLC.
- Combining ADCs, vaccines, and cell therapies may overcome immune escape and treatment resistance.
- Biomarker-guided immunotherapy selection can improve clinical outcomes and patient survival.

## Data Availability

The original contributions presented in the study are included in the article/supplementary material. Further inquiries can be directed to the corresponding author.

## Glossary

ADC: Antibody–Drug Conjugate
AI: Artificial Intelligence
ALK: Anaplastic Lymphoma Kinase
APC: Antigen-Presenting Cell
BRAF: v-Raf Murine Sarcoma Viral Oncogene Homolog B
CAF: Cancer-Associated Fibroblast
CAR: Chimeric Antigen Receptor
CAR-NK: Chimeric Antigen Receptor Natural Killer Cell
CAR-T: Chimeric Antigen Receptor T Cell
CEA: Carcinoembryonic Antigen
CEACAM5: Carcinoembryonic Antigen-Related Cell Adhesion Molecule 5
CRISPR: Clustered Regularly Interspaced Short Palindromic Repeats
CRS: Cytokine Release Syndrome
CTLA-4: Cytotoxic T-Lymphocyte-Associated Protein 4
ctDNA: Circulating Tumor DNA
DC: Dendritic Cell
EGFR: Epidermal Growth Factor Receptor
FDA: Food and Drug Administration
HER2: Human Epidermal Growth Factor Receptor 2
HER3: Human Epidermal Growth Factor Receptor 3
HLA: Human Leukocyte Antigen
ICI: Immune Checkpoint Inhibitor
IFN-γ: Interferon Gamma
IL: Interleukin
KRAS: Kirsten Rat Sarcoma Viral Oncogene Homolog
LAG-3: Lymphocyte Activation Gene-3
MDSC: Myeloid-Derived Suppressor Cell
MET: Mesenchymal–Epithelial Transition Factor
MHC: Major Histocompatibility Complex
mRNA: Messenger Ribonucleic Acid
MUC1: Mucin 1
NGS: Next-Generation Sequencing
NK: Natural Killer
NSCLC: Non-Small Cell Lung Cancer
ORR: Objective Response Rate
OS: Overall Survival
PD-1: Programmed Cell Death Protein 1
PD-L1: Programmed Death Ligand 1
PFS: Progression-Free Survival
RET: Rearranged During Transfection
ROS1: ROS Proto-Oncogene 1, Receptor Tyrosine Kinase
scRNA-seq: Single-Cell RNA Sequencing
TAM: Tumor-Associated Macrophage
TCR: T-Cell Receptor
TCR-T: T-Cell Receptor-Engineered T Cell
TGF-β: Transforming Growth Factor Beta
TIL: Tumor-Infiltrating Lymphocyte
TIM-3: T-Cell Immunoglobulin and Mucin-Domain Containing-3
TKI: Tyrosine Kinase Inhibitor
TMB: Tumor Mutational Burden
TME: Tumor Microenvironment
Treg: Regulatory T Cell
TROP2: Trophoblast Cell Surface Antigen 2
VEGF: Vascular Endothelial Growth Factor.
